# Quantitative Analysis of Pork and Chicken Products by Droplet Digital PCR

**DOI:** 10.1155/2014/810209

**Published:** 2014-08-27

**Authors:** Yicun Cai, Xiang Li, Rong Lv, Jielin Yang, Jian Li, Yuping He, Liangwen Pan

**Affiliations:** Technical Center for Animal, Plant and Food Inspection and Quarantine, Shanghai Entry-Exit Inspection and Quarantine Bureau of China, No. 1208, Minsheng Road, Pudong New Area, Shanghai 200135, China

## Abstract

In this project, a highly precise quantitative method based on the digital polymerase chain reaction (dPCR) technique was developed to determine the weight of pork and chicken in meat products. Real-time quantitative polymerase chain reaction (qPCR) is currently used for quantitative molecular analysis of the presence of species-specific DNAs in meat products. However, it is limited in amplification efficiency and relies on standard curves based Ct values, detecting and quantifying low copy number target DNA, as in some complex mixture meat products. By using the dPCR method, we find the relationships between the raw meat weight and DNA weight and between the DNA weight and DNA copy number were both close to linear. This enabled us to establish formulae to calculate the raw meat weight based on the DNA copy number. The accuracy and applicability of this method were tested and verified using samples of pork and chicken powder mixed in known proportions. Quantitative analysis indicated that dPCR is highly precise in quantifying pork and chicken in meat products and therefore has the potential to be used in routine analysis by government regulators and quality control departments of commercial food and feed enterprises.

## 1. Introduction

In 2013, the horse meat adulteration scandal [1, 2] swept across Europe, a region which is considered to hold the highest food safety standards worldwide. In this scandal, meat foods advertised as containing beef were found to contain undeclared meat, such as horse meat. In some cases, as much as 100% of the meat content was other undeclared meats. Due to the similar textures and processing technologies of different meat products, it is difficult for consumers to identify their authenticity, not to mention the precise proportion of meats within a given product. The temptation for huge profit and quick gain has made meat food adulteration a persistent problem where high quality meat products are replaced with cheaper and inferior counterparts; therefore, state or government quality inspection programs are responsible for ensuring the safety of meat products. Yet, reliable qualitative and quantitative detection methods need to be researched and developed to make this task easier.

Compared to conventional polymerase chain reaction (PCR) [3, 4] which has been widely used as a qualitative method to detect whether species-specific target DNAs exist in food and feed samples, real-time polymerase chain reaction (qPCR) [5, 6] has the advantage not only to detect but also to quantify the relationship between the cycle threshold (Ct) values and the initial DNA template concentration. Yet, qPCR technology still has several issues [7, 8] that directly affect the accuracy of quantitative analysis, including the PCR amplification efficiency, the use of standard curves based on Ct values, the high background produced by nontarget DNA samples, and problems with the selection of a suitable reference material.

Digital PCR (dPCR) is a novel method for precise quantification of nucleic acids [9, 10], which utilizes a limiting dilution analysis and Poisson distribution analysis to enable the absolute quantification of target DNA copy number [11]. Digital PCR uses a similar amplification reaction system as a standard qPCR system. A droplet generator is used to partition each dPCR reaction mix into 20,000 nanoliter-sized droplets. Each droplet contains zero (negative), one, or more copies of the target DNA (positive). After a conventional PCR procedure, the total number of template-positive or -negative individual droplets is counted and recorded by a droplet reader. Finally, the original absolute target DNA copy number (copies/*μ*L) of the samples can be directly calculated by following the Poisson distribution law.

As a refinement of conventional qPCR, dPCR has the potential to allow for more accurate and sensitive measurement of the target DNA copy number, especially for low concentration samples, high background samples, and composite samples. As a result, dPCR has been applied in a wide range of areas including quantitative gene expression analysis [12], single nucleotide polymorphism analysis [13], genotyping [14], rare variant and copy number variation detection, pathogen detection [15], drug resistance research [16], and noncoding RNA research [17].

Here, we present a meat product weight measurement system based on dPCR technology for the accurate detection and quantification of specific nucleic acids in chicken or pork. Using this simple amplification and calculation procedure, the weight of meat in the sample can be accurately quantified. This is the first time that the dPCR technique has been utilized to quantify meat products.

## 2. Materials and Methods

### 2.1. Meat Sample Preparation

Fresh lean meat and commercially available products were obtained from the local supermarket. Fresh pork (*Sus scrofa*), chicken (*Gallus gallus*), and commercial products were separately minced, dried in a baking oven (UFE500AO; Memeert, Germany) at 80°C for 72 h, and then minced to a superfine powder in liquid nitrogen using a Freezer Mixer (6850 freezer/mill; SPEX SamplePrep, USA). The mixed samples of pork and chicken powers with known composition (from 90% to 10%) were used to verify the validity and sensitivity of the method. In order to guarantee that the extracted DNA accurately represents the proportion of different meats, the Freezer Mixer was used to grind the mixtures evenly to ensure complete mixing. The commercially available products were used to test the applicability of this method.

### 2.2. DNA Extraction

For all samples, genomic DNA was extracted from 100 mg powder using the phenol/chloroform method [19]. Briefly speaking, 800 *μ*L histiocyte lysis buffer (Tiangen, China) with 100 *μ*g proteinase K (Tiangen, China) was added to each sample, vortexed, and incubated at 65°C for 60 min with occasional vigorous shaking; an equal volume of phenol/chloroform was added, mixed, and centrifuged at 12000 rpm for 10 min. The aqueous (upper) layer was transferred to a clean tube; an equal volume of chloroform was added, mixed, and then centrifuged for 5 min at 12000 rpm. The aqueous (upper) layer was transferred to a clean tube; a one-tenth volume of 3 M Na acetate (pH 5.2) and two volumes of ice-cold EtOH (100%) were added, mixed, and incubated at −20°C for 30 min and then centrifuged at 12000 rpm for 30 min at 4°C. The supernatant was removed and the DNA pellet was washed twice with 75% EtOH and centrifuged at 12000 rpm for 2 min at 4°C; the supernatant was removed and the pellet was air-dried for 30 min at room temperature, resuspended in 100 *μ*L ddH_2_O, and stored at −20°C.

### 2.3. Primers and Probes

To detect pork and chicken, the* Sus Scrofa* beta-actin (*ACTB*) gene (GenBank accession number: DQ452569) [20] and* Gallus gallus* transforming growth factor beta-3 (*TGFB3*) gene (GenBank accession number: AY685072) [18] were selected as the target detection sequences, respectively, as previously described [21–23]. Online tools supported by NCBI were used for sequence search and alignment. Primers and probes were designed using Primer Express Software version 3.0 supported by Applied Biosystems (ABI, Foster City, CA, USA). All of the selected primers and probes passed a specificity and homology evaluation by BLAST searches against the entire GenBank database. The nucleotide sequences of the primers and probes used in this study were designed to meet optimal conditions for dPCR [24]. A probe labeled with the fluorophore VIC (ABI, Foster City, CA, USA) and minor groove binder (MGB) quencher was used to detect pork. The FAM fluorophore and Black Hole Quencher (BHQ) were used to detect chicken (Table 1).

### 2.4. Specificity

In order to verify the specificity of the dPCR system (including the primers and probes), DNA from a wide range of animal samples was isolated and tested by dPCR system.

### 2.5. Digital PCR Procedure

Each 20 *μ*L reaction mixture was prepared as follows: 1.8 *μ*L of each primer (final concentration, 900 nM), 0.5 *μ*L probe (final concentration, 250 nM), and 10 *μ*L ddPCR Master Mix (Bio-Rad, Hercules, CA, USA) were mixed, and then 4 *μ*L (40-fold diluted from the original DNA extraction sample) of template DNA and 1.9 *μ*L of nuclease- and protease-free water (ThermoScientific, Salt Lake City, UT, USA) were added. A Bio-Rad QX100 ddPCR droplet generator (Bio-Rad) was used to divide the 20 *μ*L mixture into approximately 20000 droplets, with the target DNA segments and PCR reagents being randomly distributed among the droplets. Conventional PCR was performed using a T100 Thermal Cycler (Bio-Rad) according to the following cycling protocol: enzyme activation for 10 min at 95°C, followed by 40 cycles of 30 sec denaturation at 94°C; 1 min annealing and extension at 60°C, followed by enzyme inactivation at 98°C for 10 min and hold at 4°C (according to the manufacturer's instructions). After PCR amplification, the droplet reader determines which droplets contain the target DNA amplicon and which do not. The software then calculates the concentration of the target DNA in copies per microliter from the fraction of positive reactions using Poisson distribution analysis.

### 2.6. Standard Curve Generation

A series of meat powders were accurately weighed using a precision electronic balance (BSA224s; Sartorius, Germany). Genomic DNA was extracted and the DNA concentration of each sample was measured using a NanoVue spectrophotometer (GE Healthcare, Little Chalfont, Buckinghamshire, UK). Ten different samples of pork and chicken powder (equally distributed in weight from 10 mg to 100 mg, three replicates per weight) and a nontemplate control (NTC) were analyzed by dPCR. The correlation coefficient of (*R* ^2^) for the weight of the meat powder and the DNA concentration was calculated using Excel (Microsoft Office 2007; Redmond, WA, USA).

## 3. Results and Discussion

### 3.1. Specificity

We chose single copy nuclear genes that are expressed at relatively stable levels in different cell types as detection targets [25]. First, BlastN searches (http://blast.ncbi.nlm.nih.gov/Blast.cgi) of the entire NCBI genome database were used to validate the specificity of the primers, probes, and PCR amplicons; all of the pork- and chicken-specific PCR primers, probes, and PCR amplicons bore a high level of species specificity. In practice, a broad range of DNA samples from different animals were isolated and tested as templates for the PCR procedure: cattle (*Bos taurus*), donkey (*Equus asinus*), sheep (*Ovis aries*), goat (*Capra hircus*), horse (*Equus caballus*), elk (*Cervus canadensis*), buffalo (*Bubalis bubalus*), rabbit (*Oryctolagus cuniculus*), duck (*Anas platyrhynchos*), goose (*Anser domesticus*), turkey (*Meleagris gallopavo*), ostrich (*Struthio camelus*), pigeon (*Columba livia*), quail (*Coturnix coturnix*), pheasant (*Phasianus colchicus*), rhesus monkey (*Macaca mulatta*), mice (*Mus musculus*), rat (*Rattus norvegicus*), goldfish (*Carassius auratus*), carp (*Cyprinus carpio*), and trout (*Oncorhynchus mykiss*). Cross amplification from other species was not observed for any primer/probe combination. Therefore, the possibility of cross amplification was excluded from a theoretical and practical perspective.

### 3.2. DNA Extraction Efficiency

**Figure 1:**
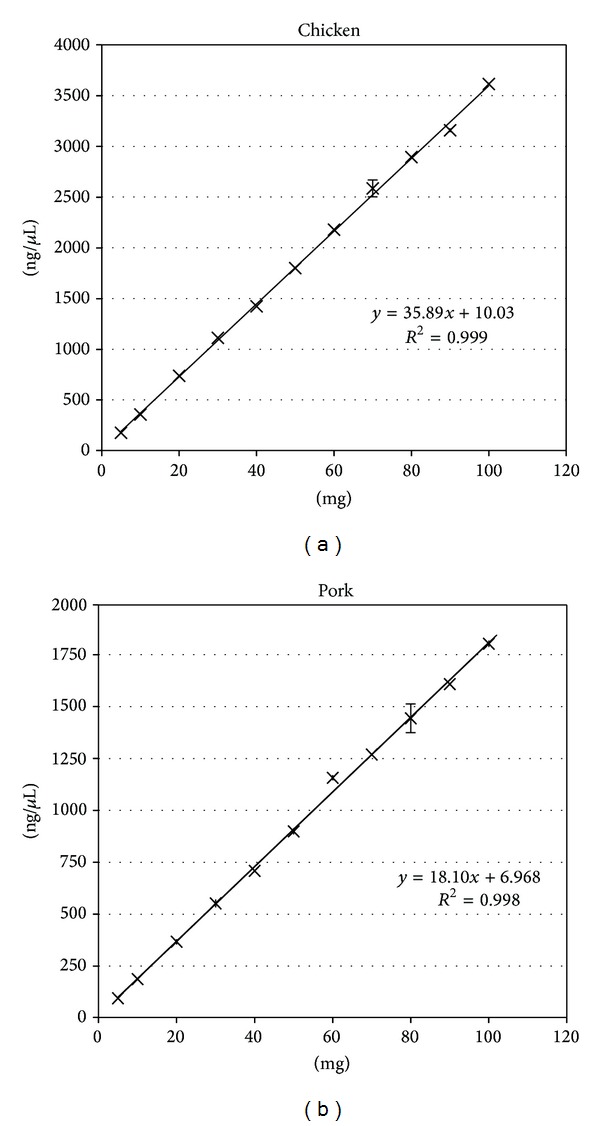
Linear relationship between meat quantity (mg) and nucleic acid (ng) content. The efficiency of extracting genomic DNA from chicken and pork was confirmed within the dynamic range. After accurate weighing and DNA extraction, the nucleic acid (ng) content of three replicates for each sample was measured using a NanoVue spectrophotometer. The correlation coefficient (*R* ^2^) for the initial sample weight (mg) and nucleic acid (ng) content was 0.999 for chicken and 0.998 for pork.

Due to the varying and complex definition of meat (fat, skin, internal organs, and so on) in food and feed products, we used fresh lean meat (chicken breast and pork loin) as the standard specimens to extract nucleic acids in this experiment to help minimize the effect of variation in the quality of the raw meat. In order to establish the relationship between the weight of meat powder (mg) and the corresponding amount of nucleic acid (ng), DNA was extracted from each meat powder sample by proteinase K digestion, phenol/chloroform extraction, and EtOH (100%) precipitation. Ten different weights of pork and chicken reference samples (equally distributed over the range from 10 mg to 100 mg, three replicates per weight) and a NTC sample were extracted. The concentration of each DNA sample was measured using a NanoVue spectrophotometer. In three independent experiments, a linear relationship was observed between the raw meat weight (mg) and the corresponding amount of extracted nucleic acid (ng). The correlation coefficient (*R* ^2^) was 0.999 for chicken (Figure 1(a)) and 0.998 for pork (Figure 1(b)). These findings indicate that, within the range between 10 mg to 100 mg raw meat powder, the amount of genomic DNA extracted had an approximately linear relationship with the weight of both types of raw meat.

### 3.3. Specific Target DNA Detection by dPCR

**Figure 2:**
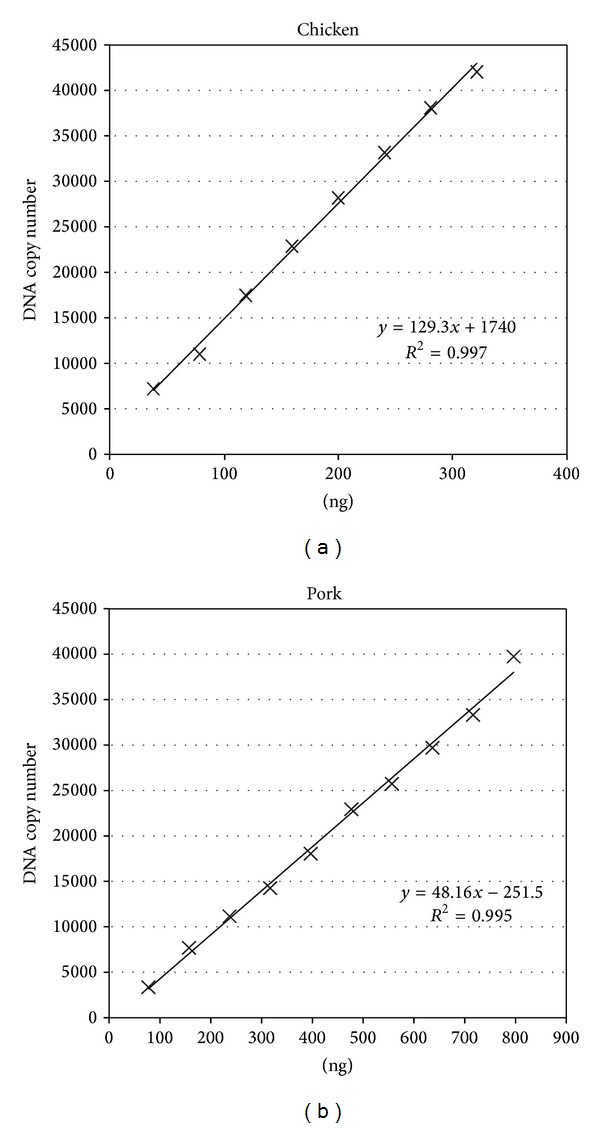
Linear relationship between nucleic acid content (ng) and the target DNA copy number. A linear relationship between the quantity of nucleic acid and the target DNA copy number was confirmed by dPCR. DNA samples of known concentration were analyzed by dPCR. Each detection point is the average of triplicate samples from three independent experiments. All experimental data met the quality requirements for dPCR. The correlation coefficient (*R* ^2^) for the DNA quantity and the DNA copy number was 0.997 for chicken and 0.995 for pork.

In order to explore whether a linear relationship exists between the weight of nucleic acid and the species-specific target DNA copy number, the serially diluted meat DNA samples and a NTC sample were analyzed by dPCR. The dPCR assays were performed on chicken samples containing 40 ng to 320 ng DNA and pork samples containing 80 ng to 800 ng DNA. The maximum concentrations were determined by the detection limit of the dPCR instrument. Each data point was collected based on three replicates per sample in three independent experiments. During the dPCR process, at least 15000 droplets were obtained for each reaction, in compliance with the requirements for absolute quantification. The correlation coefficients (*R* ^2^) for the nucleic acid weight (ng) and the chicken- or pork-specific DNA copy number were 0.997 and 0.995, respectively (Figures 2(a) and 2(b)). Results from these experiments indicate that, within the range of 40 ng to 320 ng for chicken (Figure 2(a)) and 80 ng to 800 ng for pork (Figure 2(b)), relationships between the nucleic acid weight and specific target DNA copy number were approximately linear. In this step, we found that the width of the linear dynamic range is not more than five orders of magnitude. Compared with the qPCR, dPCR offers a narrow dynamic range as described in the previous articles [24, 26]. But after appropriate dilution, the dynamic range will cover the whole range of quantification needed.

### 3.4. Establishment of Quantitative Formulae

Results from the experiments confirmed two linear relationships: one between the raw meat weight and nucleic acid weight and the other between the nucleic acid weight and specific target DNA copy number. These correlations were essential for establishing the formulae to calculate the raw meat weight. We utilized the nucleic acid weight as an intermediate value to establish the following formulae for calculating the original raw meat weight from the specific DNA copy number: chicken, *M*_chicken_ = 0.04*C* − 4, and pork, *M*_pork_ = 0.2*C* + 2.5, where *C* is the copy number (copies/*μ*L) and *M* the raw meat weight (mg).

### 3.5. Analysis of Samples of Known Concentration

Mixed meat products often appear in food products and various industrial applications. However, the DNA extraction process could be affected by numerous factors, such as the tissue composition, sample treatment, DNA degradation, and even pipetting errors. Therefore, the species-specific DNA may not truly represent the actual weight proportion of meat(s) in the product. In order to further demonstrate the overall accuracy and applicability of our method, nine mixed meat samples of known composition were used to verify the quantification method. DNA was extracted in duplicate from each mixed meat sample using the same method. Each DNA sample was diluted 40-fold and 4 *μ*L of each sample was analyzed in triplicate in the same dPCR experiment; the data was expressed as the average value. The original meat weight was calculated using the two formulae above. Examined through three different independent experiments, the dPCR technology had a high consistency and reproducibility. More importantly, the final quantitative results for the mixed pork and chicken samples were similar to the true raw meat weights (Table 2). Compared to the qPCR quantification method [18], most of the measured meat weights in this study had a low level of deviation, indicating that the dPCR assay is highly accurate. The variations in the values obtained using dPCR may be due to the heterogeneity of the raw meat samples or artificial operator errors. Therefore, the sample processing steps of dry powder generation, DNA extraction, and sampling uniformity are critically important to the test procedure. Complete drying and full grinding of the test samples are the most basic requirements for accurate quantification.

### 3.6. Analysis of Commercial Samples

A total of 11 commercially available products (Table 3) were collected and analyzed by dPCR quantification system to determine the proportion of the pork or chicken. The result of the experiment validates that the system has good practicability.

## 4. Conclusions

Through a well-designed experiment, this study has demonstrated that the dPCR technique can be used to accurately quantify the weight of specific meats in meat products. A total of 11 commercially available meat products were used to prove the applicability of this system. Some influential factors during the quantification procedure were taken into account. We chose fresh lean meat to guarantee the consistency of DNA content and chose stable (relatively) expression DNA sequence as the detection target, so the quantification accuracy will be guaranteed. Satisfyingly, the relationships between the raw meat weight and DNA weight and between DNA weight and DNA copy number were both close to linear for both pork and chicken. This enabled us to establish formulae to calculate the raw meat weight based on the DNA copy number. This technique has the potential to provide a convenient way to quantify the meat content of foods or feed. No optimization steps were required during the course of the experiment. The primers and probes from routine qPCR systems can be used directly in a dPCR quantification system. We investigated the accuracy of this technique and found that dPCR could achieve a linear dynamic range for absolute quantification of DNA. In each dPCR reaction, nearly 15000 effective droplets were generated, detected, and analyzed, which ensured the accuracy of the quantification method. Additionally, calculations involved in dPCR are based on absolute data instead of relative data (such as Ct values) and do not require standard curves or reference materials, which improve the accuracy of quantification. This work is the first to demonstrate how to apply the dPCR technology to quantify pork and chicken in meat products. Suitable for routine analysis, this method has the potential to be adapted to quantifying meat of various species. However, a number of technical flaws, such as narrow dynamic range and time consuming nature of the assay, remain to be solved before dPCR can be widely adopted for the routine quantification of the meat content in food and feed products.

## Figures and Tables

**Table 1:** Primer and probe sequences for quantitative dPCR assays.

Primer/probe	Sequence/labeling	GenBank accession number
Sus-ACTB-97bp-F	CGTAGGTGCACAGTAGGTCTGAC	Beta-Actin gene
Sus-ACTB-97bp-R	GGCCAGACTGGGGACATG	DQ452569
Sus-ACTB-97bp-P	VIC-CCAGGTCGGGGAGTC-MGB	

Gallus-TGFB3-129bp-F	GGCTGCAAGTCACCGTGGTA	TGFB3 gene
Gallus-TGFB3-129bp-R	CCGCTAGCCAGAAGCTCAGC	AY685072
Gallus-TGFB3-129bp-P	FAM-CAGGAGCCACGTGAGCAGCACAG-BHQ [18]	

**Table 2:** The results of quantification of the samples with known concentrations.

	Pork true (mg)	Pork measure (mg)	Pork deviation	Chicken true (mg)	Chicken measure (mg)	Chicken deviation
Sample 1	90	97.50	8.33%	10	8.72	−12.80%
Sample 2	85	84.43	−0.67%	15	16.85	12.36%
Sample 3	70	73.63	5.19%	30	28.59	−4.71%
Sample 4	65	63.50	−2.31%	35	32.33	−7.62%
Sample 5	50	52.37	4.73%	50	49.00	−2.00%
Sample 6	45	46.90	4.22%	55	57.53	4.61%
Sample 7	30	28.83	−3.89%	70	64.25	−8.21%
Sample 8	25	22.30	−10.80%	75	76.81	2.42%
Sample 9	10	11.70	17.00%	90	94.11	4.56%

**Table 3:** Samples from the local supermarket were analyzed.

Sample	Chicken (%)	Pork (%)
Chicken ham sausage	3.8	0.0
Pork ham sausage	0.0	2.6
Beef ham sausage	0.0	0.0
Minced chicken	48.6	0.0
Minced pork	0.0	35.1
Chicken vegetable dog food	25.1	0.0
Pork spam	0.0	3.2
Canned stewed pork	0.0	41.6
Beef vegetable dog food	0.0	0.0
Fish cat food	0.0	0.0
Chicken flavor	0.0	0.0
